# DEMENTIA CAREGIVER SUPPORT IN THE RURAL, FRONTIER, AND TRIBAL AREAS OF UTAH

**DOI:** 10.1093/geroni/igae098.1229

**Published:** 2024-12-31

**Authors:** Kate Nederostek, Kristy Russell

**Affiliations:** Utah Department of Health and Human Services, Salt Lake City, Utah, United States; Utah Department of Health and Human Services, Salt Lake City, Utah, United States

## Abstract

More than three million people call the Beehive state home. Rapid growth (40% in the last 20 years) and Utah’s unique landscape present challenges when it comes to coordination of services. Made up of 29 counties covering 84,000 square miles, Utah is the 13th largest state by area. Approximately three-fourths of Utah’s population lives in five contiguous counties, often referred to as the Wasatch Front. However, 12 of Utah’s counties are considered rural (six to 100 persons per square mile) and 12 counties are considered frontier (six or fewer persons per square mile). Utah’s statewide dementia efforts are funded by the state legislature (~$430,000/year) and currently receive no additional federal funds. Historically, the federally supported organizations that serve frontier and rural communities in Utah receive less funding due to their lower population density. To better serve their communities, the Alzheimer’s Disease and Related Dementias (ADRD) Program and Family Caregiver Support Program, offered through the Area Agencies on Aging (AAAs), have combined forces. The ADRD Program assists each AAA in providing education, support, materials, and programming to those living with dementia and their caregivers. This collaboration has allowed an extension of programs statewide, providing an opportunity for those in rural and frontier areas to participate. Relationships and collaborative interest are growing with the Utah Indian Health Advisory Board. Utah’s ADRD Program continues to work through barriers and to tailor educational opportunities to the needs of each tribal area.

